# Long non-coding RNA DLEU1 promotes malignancy of breast cancer by acting as an indispensable coactivator for HIF-1α-induced transcription of CKAP2

**DOI:** 10.1038/s41419-022-04880-z

**Published:** 2022-07-19

**Authors:** He Nan Ma, Hai Jun Chen, Ji Quan Liu, Wen Tao Li

**Affiliations:** Department of Breast Surgery, Henan Provincial People’s Hospital, Zhengzhou University People’s Hospital, Henan University People’s Hospital, Zhengzhou, 450003 Henan Province China

**Keywords:** Cell migration, Cancer

## Abstract

Earlier studies have suggested deleted in lymphocytic leukemia 1 (DLEU1), a long non-coding RNA, is a prognostic biomarker for breast cancer. Here we explored the malignant behaviors and underlying mechanisms regulated by DLEU1 in breast cancer. We demonstrated that up-regulation of DLEU1 was detected in breast cancer tissues and cells, particularly in tumors of higher malignancy. DLEU1 knockdown inhibited the growth and the motility of breast cancer cells. Mechanistically, DLEU1 interacted with HIF-1α to collectively activate the transcription of CKAP2. By activating ERK and STAT3 signaling, CKAP2 essentially mediated the pro-tumor activities of DLEU1. In vivo, depletion of DLEU1 inhibited xenograft growth and metastasis of breast cancer cells. Therefore, DLEU1, by acting as a coactivator for HIF-1α, up-regulates CKAP2 expression and promotes malignancy of breast cancer. Targeting DLEU1, HIF-1α, or CKAP2 may thus benefit breast cancer treatment.

## Introduction

Increasing studies demonstrate aberrant expression of long non-coding RNAs (lncRNAs) in cancers, supporting their role as biomarkers for cancer diagnosis and prognosis. The lncRNA deleted in lymphocytic leukemia 1 (DLEU1) is encoded by a gene localized in chromosome 13q14.3, a chromosomal region frequently deleted in spindle cell lipoma and other hematological malignancies [1–4], suggesting it may be a tumor suppressor. However, recent studies reveal oncogenic activities of DLEU1: it is up-regulated in a panel of human cancers, correlates with worse prognosis, and promotes various malignant phenotypes [5–12]. Although studies suggest the up-regulation of DLEU1 in breast cancer [9, 13], limited information is available regarding its biological effects and molecular mechanisms [11], which, therefore, becomes a focus for this study.

Through the interaction with other molecules, including DNAs, RNAs, or proteins, lncRNAs may function as a decoy, a scaffold, a guide, or an enhancer [14]. While searching for the potential interacting partner of DLEU1 using the catRAPID algorithm, we identified hypoxia-inducible factor 1α (HIF-1α), which, when heterodimerizes with the constitutively expressed β subunit, forms the transcription factor HIF, regulates various target genes, and stimulates cancer progression [15]. The potential interaction between DLEU1 and HIF-1α suggests that DLEU1 may present its oncogenic activities by interacting with HIF-1α. In addition, using JASPAR, we identified a hypoxia response element (HRE) within the promoter region of human cytoskeleton-associated protein 2 (CKAP2) gene. CKAP2, through the association with microtubules, regulates cell cycle progression and cell death. Recent studies suggest that CKAP2 is up-regulated and contributes to the malignant progression of diverse human cancers, including breast cancer [16–20]. Based on these clues, we hypothesize that DLEU1, via interacting with HIF-1α, controls the transcription and expression of CKAP2. In return, CKAP2 mediates, at least partially, the oncogenic activities of DLEU1. To test this hypothesis, we explored the interactions between DLEU1, HIF-1α, and CKAP2 in breast cancer tissues acquired from patients or database, in breast cancer cells cultured in vitro, and in in vivo xenografts or metastasis models.

## Results

### DLEU1 expression is higher in breast cancer tissues or cells, and is an indicator for tumors of higher malignancy

**Fig. 1:**
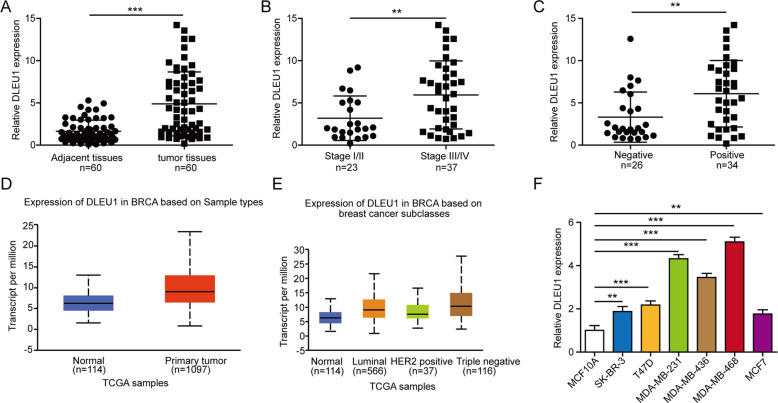
Higher expression of DLEU1 in breast cancer is an indicator for tumors of higher malignancy. The expression of DLEU in 60 pairs of breast cancer and adjacent normal breast tissues was measured by RT-PCR (**A**), between tumors of different TNM stages (*n* = 23 for stage I/II and *n* = 37 for stage III/IV) (**B**), and between tumors with negative (*n* = 26) and those with positive lymph node metastasis (*n* = 34) (**C**). DLEU1 expression was analyzed in TCGA database between primary breast tumors (*n* = 1097) and normal breast tissues (*n* = 114; *P* < 0.001) (**D**), and between tumors of different molecular subtypes, including 566 luminal, 37 HER2-positive, and 116 TNBC, and normal breast tissues (*n* = 114; *P* < 0.001 between normal and the other three subtypes, and also between TNBC and the other two groups; *P* > 0.05 between luminal and HER2-positive groups) (**E**). **F** DLEU1 expression was examined by RT-PCR in non-tumorigenic MCF10A and indicated breast cancer cells. *P < 0.05, **P < 0.01, ***P < 0.001.

An earlier study has suggested that higher DLEU1 expression is a pan-cancer marker for worse prognosis [9]. Considering the prevalence of breast cancer in clinic yet limited information available for DLEU1 significance or mechanism in this type of cancer [11, 13], we focused on breast cancer in this study. We first compared DLEU1 expression between 60 pairs of breast cancer and para-tumor normal tissues and detected its robust up-regulation in cancer tissues (Fig. 1A). Next, we analyzed the associations between DLEU1 expression level and TNM stages or the status of lymph node metastasis. Significantly higher DLEU1 expression was detected in tumors of advanced TNM stages, i.e., stage III/IV (*n* = 37) than in those of early stages (I/II, *n* = 23; Fig. 1B), and also in tumors with positive lymph node metastasis (*n* = 34) than in those without (*n* = 26; Fig. 1C). In addition to the 60 samples we collected from clinic, we performed analysis on TCGA dataset containing much larger number of samples. We found that DLEU1 expression was significantly up-regulated in primary breast cancers (*n* = 1097), when compared to normal breast tissues (*n* = 114; Fig. 1D). When stratifying breast cancer tissues into different molecular subtypes, we found that DLEU1 expression was significantly higher in triple-negative breast cancer (TNBC) tissues (*n* = 116) than in luminal (*n* = 566) or HER2-positive cancers (*n* = 37; Fig. 1E). Lastly, we measured DLEU1 expression in a panel of breast cancer cell lines of different molecular subtypes [21], including luminal MCF7 and T47D, HER2-positive SK-BR-3, and TNBC MDA-MB-231, MDA-MB-436, and MDA-MB-468 cells. When compared to the non-tumorigenic breast epithelial MCF10A cells, DLEU1 expression was elevated in all breast cancer cell lines examined but more strikingly in three TNBC cell lines (Fig. 1F). Together, the data demonstrate the elevated expression of DLEU1 in breast cancer and its association with tumors of higher malignancy, supporting its potential involvement in the pathogenesis of breast cancer.

### DLEU1 essentially controls multiple malignant behaviors of breast cancer cells

**Fig. 2:**
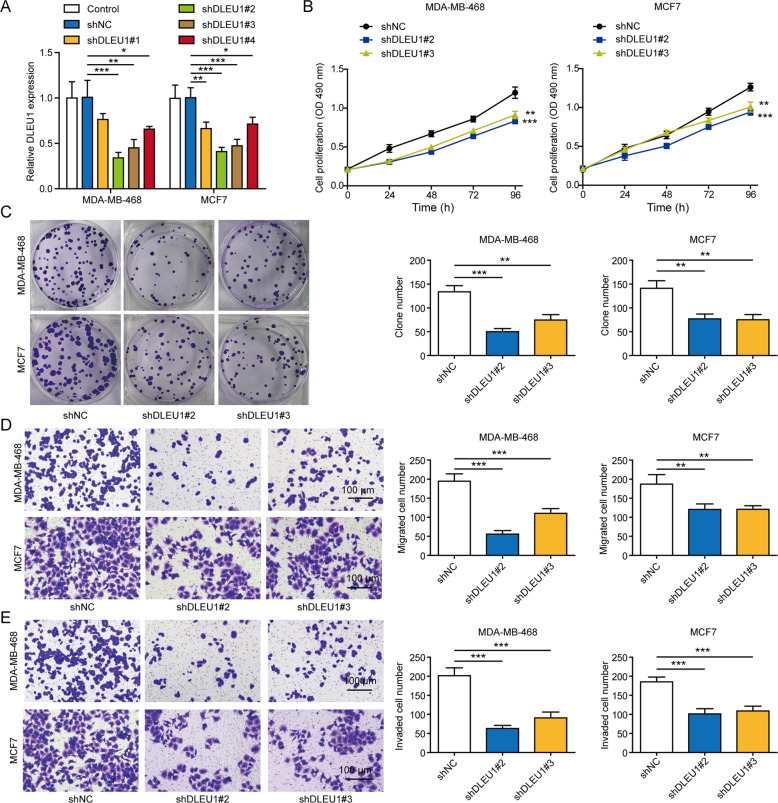
DLEU1 essentially controls various malignant behaviors of breast cancer cells. MDA-MB-468 and MCF7 cells were either not transfected (control), or stably transfected with shNC, shDLEU1#1, #2, #3, or #4. **A** DLEU1 level was examined by RT-PCR. **B** The proliferation of shNC, shDLEU1#2, or shDLEU1#3 cells was examined by MTT assay. **C** Cell proliferation was examined by colony formation assay (left), with the quantification of colony numbers on the right. The migration (**D**) and the invasion (**E**) of indicated cells were examined by Transwell assays (left), with the number of migrated/invaded cells presented on the right. *P < 0.05, **P < 0.01, ***P < 0.001.

Considering the up-regulated DLEU1 in breast cancer, we applied the loss-of-function strategy in one TNBC cell line, MDA-MB-468, and one non-TNBC cell line, MCF7. We designed four specific shRNA sequences targeting DLEU1 (shDLEU1#1 to #4). Upon stably transfecting MDA-MB-468 or MCF7, we found that shDLEU1#2 and #3 most robustly reduced endogenous DLEU1 level in these cells (Fig. 2A) and thus were used for future experiments. Next, we measured the effects of knocking down DLEU1 on multiple malignant behaviors of cancer cells and found that when compared to control shRNA (shNC)-transfected cells, shDLEU1#2 or #3 cells significantly reduced short-term cell proliferation (Fig. 2B), long-term proliferation (Fig. 2C), migration (Fig. 2D), and invasion (Fig. 2E), suggesting that DLEU1 critically controls multiple malignant phenotypes of breast cancer cells.

### Up-regulation of CKAP2 by DLEU1 is required for the pro-tumor activities of the latter

**Fig. 3:**
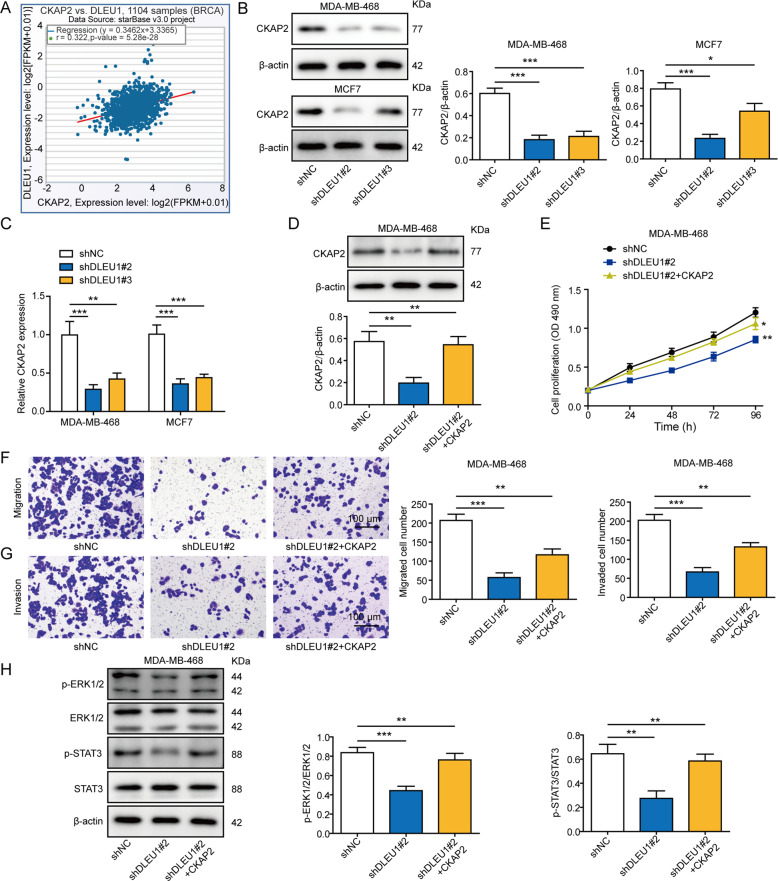
Up-regulation of CKAP2 by DLEU1 is required for the pro-tumor activities of the latter. **A** Analysis of TCGA database revealed a significantly positive correlation between DLEU1 and CKAP2 expression levels in breast cancer tissues (*n* = 1104). MDA-MB-468 and MCF7 cells were stably transfected with shNC, shDLEU1#2, or shDLEU1#3. The expression of CKAP2 was examined on the protein level by western blot (**B**) and on the mRNA level by RT-PCR (**C**). MDA-MB-468 and SK-BR-3 cells were stably transfected with shNC, shDLEU1#2, or shDLEU1#2 + CKAP2. CKAP2 protein (**D**), cell proliferation (**E**), migration (**F**), invasion (**G**), and expressions of p-ERK1/2, ERK1/2, p-STAT3, and STAT3 (**H**) were examined by western blot, MTT assay, Transwell migration, Transwell invasion, and western blot, respectively. *P < 0.05, **P < 0.01, ***P < 0.001.

To explore the molecular mechanisms underlying the pro-tumor activities of DLEU1, we resorted to TCGA database to identify molecules whose expressions are correlated with DLEU1. We found that CKAP2 expression presented a positive correlation with DLEU1 in breast cancer tissues (*n* = 1104; Fig. 3A), implying a potential regulation between these two molecules, which has not been reported. CKAP2 is a microtubule-associated protein overexpressed in various human cancers and promoting cancer proliferation and tumorigenesis [16, 17, 19, 20]. Previous studies suggested that the activation of FAK/ERK or JAK2/STAT3 signaling mediated the pro-tumor activities of CKAP2 [19, 20]. By examining CKAP2 protein (Fig. 3B) and mRNA (Fig. 3C) in shNC vs. shDLEU1#2 or #3 cells, we found that knocking down DLEU1 was sufficient to markedly reduce CKAP2 protein or mRNA levels. To assess the functional significance of CKAP2 in DLEU1-mediated malignant phenotypes, we overexpressed CKAP2 in shDLEU1#2 cells (shDLEU1#2 + CKAP2) (Fig. 3D). Overexpressing CKAP2 in shDLEU1#2 cells partly abolished shDLEU1-induced inhibition on cell proliferation (Fig. 3E), migration (Fig. 3F), or invasion (Fig. 3G). On the molecular level, we detected significant suppression of ERK (as represented by reduced p-ERK1/2) and STAT3 (as indicated by reduced p-STAT3) signaling in shDLEU1#2 cells, which was negated in shDLEU1#2 + CKAP2 cells (Fig. 3H). Collectively, these data suggest that CKAP2, by activating ERK and STAT3 signaling, critically mediates the malignant phenotypes induced by DLEU1.

In addition to the loss-of-function strategy, we also examined the impact of overexpressing DLEU1 on the malignant phenotypes of MCF7 cells. As shown in Supplemental Fig. S1, overexpressing DLEU1 (Fig. S1A) was sufficient to significantly boost the cell proliferation at 96 h after the transfection (Fig. S1B) and stimulate cell migration (Fig. S1C). On the molecular level, we detected the significant increases of CKAP2 protein (Fig. S1D) and mRNA (Fig. S1E) expression in DLEU1-overexpressing MCF7 cells, supporting the association between DLEU1-induced up-regulation of CKAP2 and the enhanced malignancy of breast cancer cells.

### DLEU1 directly interacts with but not controls the expression of HIF-1α

**Fig. 4:**
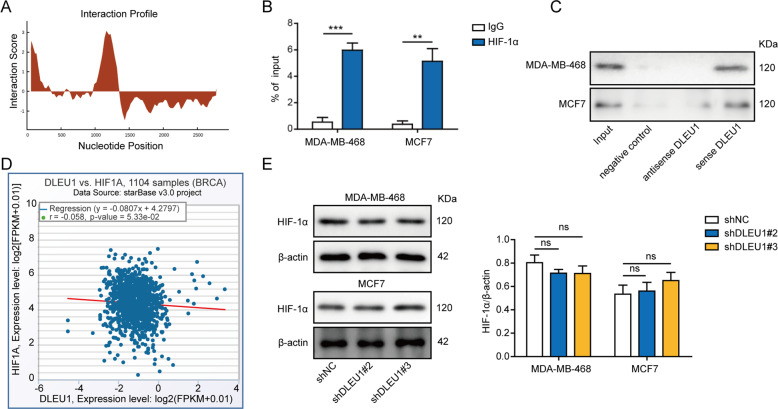
DLEU1 directly interacts with but not controls the expression of HIF-1α. **A** The interaction between DLEU1 and HIF-1α was analyzed using catRAPID algorithm. **B** The interaction between DLEU1 and HIF-1α was examined by RIP assay in MDA-MB-468 and MCF7 cells. The amount of immunoprecipitated DLEU1 was presented as a % of total input. **C** The interaction between DLEU1 and HIF-1α was examined by RNA pull-down assay in MDA-MB-468 and MCF7 cells using negative control, sense DLEU1, or antisense DLEU1 as the probe. **D** Analysis of TCGA database revealed no significant correlation between DLEU1 and HIF-1α expression levels in breast cancer tissues (*n* = 1104). **E** MDA-MB-468 and MCF7 cells were stably transfected with shNC, shDLEU1#2, or shDLEU1#3. HIF-1α level was detected by western blot. **P < 0.01, ***P < 0.001, and ns not significant.

Cumulative studies have revealed several regulatory mechanisms for lncRNA, including recruiting chromatin-remodeling complexes to achieve epigenetic regulation of chromatin, modulating gene transcription as co-factors for transcriptional factors, and processing mRNAs at different post-transcriptional steps [22]. To understand the mechanisms by which DLEU1 up-regulated CKAP2, we performed catRAPID analysis and found HIF-1α as a putative interacting protein with DLEU1 (Fig. 4A). HIF-1α is a subunit of the HIF-1 transcription factor, a master regulator of hypoxia, and an oncogenic protein promoting malignant phenotypes of breast cancer and other human cancers [15]. The direct interaction between DLEU1 and HIF-1α was confirmed in both MDA-MB-468 and MCF7 cells using RIP (Fig. 4B) and RNA pull-down (Fig. 4C) assays. TCGA analysis of 1104 breast cancer samples revealed no significant correlation between the expression levels of DLEU1 and HIF-1α (Fig. 4D). Consistently, HIF-1α expression was not significantly changed between shDLEU1#2 or #3 cells and shNC cells (Fig. 4E). These data support that DLEU1 directly interacts with HIF-1α, but not controls its expression.

### DLEU1 is a co-factor required for HIF-1α-induced transcription of CKAP2

**Fig. 5:**
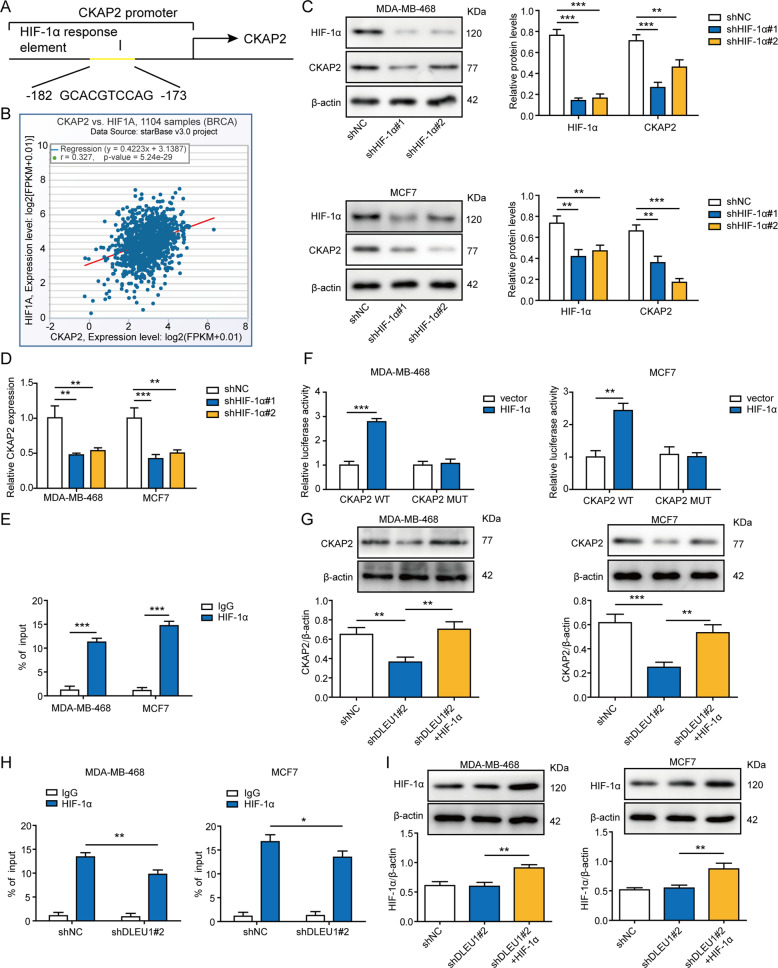
DLEU1 is a co-factor required for HIF-1α-activated transcription of CKAP2. **A** JASPAR analysis identified a HRE in the promoter region (−182 to −173 nucleotides upstream of the transcription start site) of human CKAP2 gene. **B** Analysis of TCGA database revealed a significantly positive correlation between CKAP2 and HIF-1α expression levels in breast cancer tissues (*n* = 1104). MDA-MB-468 and MCF7 cells were stably transfected with shNC, shHIF-1α#1, or shHIF-1α#2. The expressions of HIF-1α and CKAP2 were examined on the protein levels by western blot (**C**) and on the mRNA levels by RT-PCR (**D**). **E** The binding of HIF-1α to CKAP2 gene was examined by ChIP assay using control IgG or anti-HIF-1α antibody in MDA-MB-468 and MCF7 cells. **F** Wildtype (WT) HRE from CKAP2 gene was mutated (MUT). WT or MUT sequence was cloned into luciferase reporter construct, co-transfected into target cells with control or HIF-1α-overexpressing plasmid, and examined for the luciferase activity. MDA-MB-468 and MCF7 cells were stably transfected with shNC, shDLEU#2, or shDLEU#2+HIF-1α. **G** The expression of CKAP2 was examined on the protein levels by western blot. **H** The binding of HIF-1α to CKAP2 gene was examined by ChIP assay using control IgG or anti-HIF-1α antibody in indicated MDA-MB-468 and MCF7 cells. **I** Western blot was employed to detect the protein level of HIF-1α. *P < 0.05, **P < 0.01, ***P < 0.001.

The direct interaction between DLEU1 and HIF-1α suggests that DLEU1 may function as a co-factor and regulate HIF-1α-controlled transcription of target genes. Although no previous study has demonstrated CKAP2 as a direct target for HIF-1α, analysis of JASPAR database revealed a HRE within the promoter of human CKAP2 gene (Fig. 5A). TCGA analysis of 1104 breast cancer samples showed a significant positive correlation between HIF-1α and CKAP2 expression levels (Fig. 5B). Consistently, when knocking down endogenous HIF-1α using two different shRNA constructs (shHIF-1α#1 and #2) in both cells, we detected significant reduction of CKAP2 on the protein (Fig. 5C) as well as the mRNA (Fig. 5D) levels. ChIP assay revealed the direct binding of HIF-1α to the promoter of CKAP2 gene in both MDA-MB-468 and MCF7 cells (Fig. 5E). Next, we mutated the HRE (MUT) and cloned either wildtype (WT) or MUT sequence into luciferase reporter construct. By reporter assay, we found that HIF-1α specifically enhanced the luciferase activity driven by WT, but not by MUT sequence (Fig. 5F). These data indicate CKAP2 is a direct transcriptional target for HIF-1α in breast cancer cells. Next, we examined the impact of DLEU1 on HIF-1α-induced transcription of CKAP2. When overexpressing HIF-1α in shDLEU1#2 cells (shDLEU1#2+HIF-1α), we found that shDLEU1-induced reduction of CKAP2 was reversed (Fig. 5G). ChIP assay showed that the binding of HIF-1α to CKAP2 promoter was significantly inhibited in shDLEU1#2 cells (Fig. 5H), suggesting that the transcriptional activation of CKAP2 by HIF-1α requires DLEU1. Besides, HIF-1α overexpression significantly increased the level of itself (Fig. 5I). Luciferase assay showed that shDLEU1#2 potently reduced reporter activity driven by CKAP2 promoter, overexpressing HIF-1α in shDLEU1#2 cells (shDLEU1#2+HIF-1α) not only reversed shDLEU1#2-induced inhibition of luciferase reporter activity but further boosted to a level significantly higher than that observed in shNC cells (Fig. S2A). To examine the effect of DLEU1 on the transcription of CKAP2, we treated shNC, shDLEU1#2, and shDLEU1#2+HIF-1α cells (both MDA-MB-468 and MCF7) with actinomycin D to block *de novo* transcription. Our data showed that knocking down DLEU1 alone robustly accelerated the degradation of endogenous CKAP2 mRNA, and this effect was abolished when HIF-1α was overexpressed (Fig. S2B).

### HIF-1α critically controls the pro-tumor activities of DLEU1

**Fig. 6:**
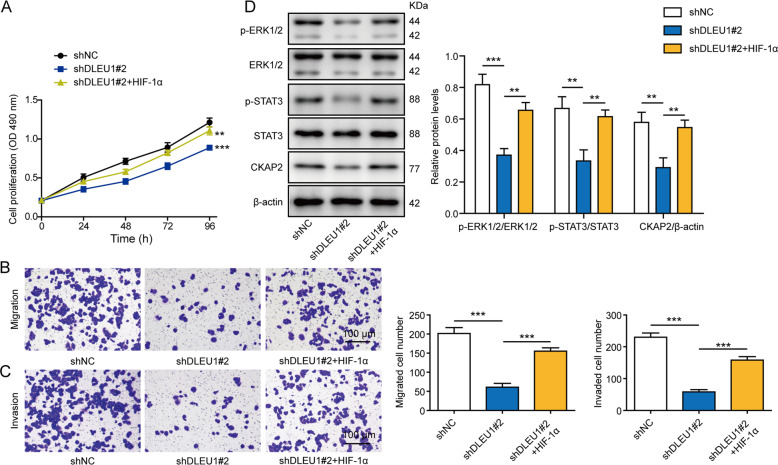
HIF-1α critically controls the pro-tumor activities of DLEU1. MDA-MB-468 cells were stably transfected with shNC, shDLEU#2, or shDLEU#2+HIF-1α. Cell proliferation (**A**), migration (**B**), invasion (**C**), and expressions of p-ERK1/2, ERK1/2, p-STAT3, and STAT3 (**D**) were examined by MTT assay, Transwell migration, Transwell invasion, and western blot, respectively. **P < 0.01, ***P < 0.001.

Upon identifying the requirement of both DLEU1 and HIF-1α for CKAP2 transcription, we examined the functional significance of HIF-1α on malignant phenotypes regulated by DLEU1. By comparing shDLEU1#2 cells with shDLEU1#2+HIF-1α cells, we found that similar to impacts presented by overexpressing CKAP2, HIF-1α was sufficient to reverse shDLEU1-induced inhibition on cell proliferation (Fig. 6A), migration (Fig. 6B), invasion (Fig. 6C), activation of ERK and STAT3 signaling, or CKAP2 expression (Fig. 6D), suggesting that by up-regulating CKAP2, HIF-1α critically mediates DLEU1-induced malignant behaviors of breast cancer cells.

### Targeting DLEU1 inhibits in vivo xenograft growth and metastasis of breast cancer cells

**Fig. 7:**
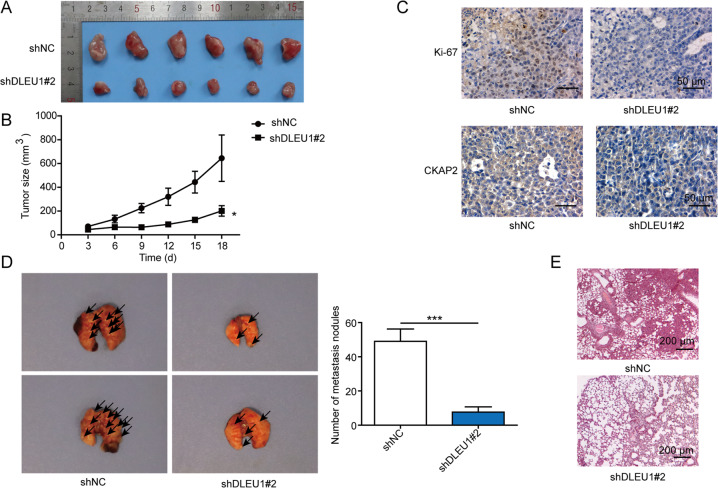
Targeting DLEU1 inhibited in vivo xenograft growth and metastasis. shNC or shDLEU1#2 cells were subcutaneously injected into nude mice (*n* = 6/group). **A** Pictures of xenografts isolated on day 18 from indicated groups. **B** Growth curve of xenografts in vivo. **C** Expressions of Ki-67 and CKAP2 were examined by immunohistochemistry from indicated xenografts. shNC or shDLEU1#2 cells were intravenously injected into nude mice (*n* = 6/group). **D** Pictures of lungs isolated on day 28 from indicated groups were shown on the left, and macroscopic metastatic nodules quantified on the right. **E** HE staining of metastatic lung tissues. *P < 0.05, ***P < 0.001.

Lastly, we assessed the impacts of targeting DLEU1 in MDA-MB-468 cells on in vivo tumorigenesis and metastasis. shDLEU1#2 MDA-MB-468 cells generated much smaller xenografts than shNC cells (Fig. 7A) and the difference was significant starting from day 10 after the inoculation (Fig. 7B). On the molecular level, we detected robust reductions of Ki-67 (a marker for cell proliferation) and CKAP2 in shDLEU1#2 xenografts (Fig. 7C). In the metastasis model, we found that shDLEU1#2 MDA-MB-468 cells generated significantly lower number of pulmonary metastasis than shNC cells (Fig. 7D, E), indicating the potency of targeting DLEU1 in inhibiting in vivo tumorigenesis and metastasis of breast cancer.

## Discussion

In the present study, we witnessed the oncogenic nature of DLEU1 in breast cancer: higher level of DLEU1 was not only significantly associated with breast cancer (in contrast to normal breast tissues), but also with those of advanced TNM stages, positive lymph node metastasis, or TNBC subtype (in contrast to luminal or HER2-positive subtype). In addition, knocking down DLEU1 robustly suppressed multiple malignant behaviors, *both* in vitro and in vivo. Mechanistically, we evidenced for the first time that DLEU1 functioned as a transcriptional co-activator for HIF-1α and was required for HIF-1α-induced transcription of CKAP2. Functionally, both CKAP2 and HIF-1α at least partially mediated the pro-tumor activities of DLEU1.

Although the deletion of ch13q14 where human DLEU1 gene is localized in certain types of cancers supports the notion that DLEU1 is a tumor suppressor [1–4], the absence of *DLEU1* gene in mouse ch13q14, together with the presence of at least several other tumor suppressor genes in this chromosomal locus, dampen the significance of loss of DLEU1 expression in tumorigenesis [23, 24]. In contrast, increasing studies focusing on DLEU1 presented convincing evidence that DLEU1 is a pan-cancer oncogene. In breast cancer, three studies have reported the up-regulation of DLEU1 in cancer tissues [9, 11, 13], consistent with our findings from clinically acquired tissue samples or from database analysis. Furthermore, we evidenced the association between higher DLEU1 expression and breast cancers demonstrating a more aggressive growth, as represented by higher TNM stages, lymph node metastasis, and TNBC subtype, suggesting that targeting DLEU1 may effectively control breast cancers of higher malignancy. In corroboration, when using luminal MCF7 and TNBC MDA-MB-468 cells as the model systems, we found that targeting DLEU1 in both cells robustly inhibited the growth and metastatic behaviors both in vitro and in vivo.

As a lncRNA, the mechanisms mediating the pro-tumor activities of DLEU1 have been revealed in different types of cancers. In many cases, DLEU1 acted as an competing endogenous RNA (ceRNA) for a microRNA, such as miR-381 in cervical cancer [7], miR-99b in bladder cancer, miR-490 in endometrial cancer [10], miR-421 in glioma [25], miR-671-5p in osteosarcoma [26], and microRNA-300 in breast cancer [11]. In a few other studies, distinct mechanisms are reported for DLEU1. For instance, Liu et al. showed that DLEU1, through the direct interaction with SMARCA1 (an essential component of the chromatin remodeling NURF complex), recruited SMARCA1 to the promoter, and activated KPNA3 transcription in colorectal cancer [8]. Du et al. reported that DLEU1 interacted with mTOR and activated PI3K/Akt/mTOR signaling in endometrial carcinoma [27]. Li et al. found that by recruiting lysine specific demethylase 1 to the promoter of KLF2, DLEU1 epigenetically suppressed its transcription in gastric cancer [5]. Here we revealed a novel mechanism underlying the oncogenic activities of DLEU1, that is, by functioning as an indispensable co-activator for HIF-1α and promoting the transcription of CKAP2. We demonstrated the direct interaction between DLEU1 and HIF-1α in breast cancer cells, the impact of knocking down each on the expression of CKAP2, and their separate yet positive correlation with CKAP2 level in breast cancer. In contrast, we did not detect any correlation between DLEU1 and HIF-1α levels in breast cancer or the effect of shDLEU1 in altering HIF-1α expression, suggesting that their crosstalk is through physical interaction, but not expressional regulation.

CKAP2, a microtubule-associated protein and a critical regulator of cell division, has been well demonstrated to as an oncogene in different cancers, such as ovarian cancer, breast cancer, osteosarcoma, glioma, cervical cancer, and gastric cancer [16, 18–20, 28, 29]. Interestingly, human CKAP2 gene is also localized ch13q14.3, the same chromosomal region harboring human DLEU1 gene. Therefore, the expressions of these two genes could be regulated by the same mechanism, such as epigenetic modulations that have been shown to up-regulate DLEU1 in cancers [9]. In such case, HIF-1α-induced transcription of CKAP2 would add a further layer of control to fine-tune the expression of CKAP2 in cancer. Earlier studies suggest p53 or Sp1 regulates the transcription of CKAP2 [30, 31], and this is the first one linking DLEU1 or HIF-1α to CKAP2. In breast cancer, CKAP2 is revealed as a biomarker for proliferation and an independent prognostic indicator [16, 17, 32]. However, little is known on signaling pathways mediating oncogenic phenotypes of CKAP2. In this study, we detected the alterations in ERK1/2 and STAT3 activation when CKAP2 level was changed in breast cancer cells, suggesting the potential involvement of these two signaling pathways, which awaits detailed characterization in future studies.

HIF-1α is a master regulator of host defense to hypoxia and a repertoire of malignant phenotypes from tumorigenesis, metastasis, angiogenesis, stemness, and drug resistance [15]. Most HIF-related biological effects are achieved through the transcription of diversified target genes. As a result, targeting mechanisms regulating HIF-1α level or its transcriptional activity has been intensively studied as anticancer strategies [33]. In this study, we identified DLEU1 as a novel and critical co-activator for the transcriptional activation of HIF-1α on CKAP2. Although there was no expressional correlation between DLEU1 and HIF-1α in breast cancer tissues, overexpressing HIF-1α in shDLEU1 breast cancer cells was sufficient to recover the malignant phenotypes resulting from knocking down DLEU1. However, many questions remain to be addressed. First, how does DLEU-1 regulate HIF-1α-induced transcription, as a recruiter/guide, a scaffold, or an enhancer? Second, are there any cancer-related targets other than CKAP2 co-regulated by DLEU1 and HIF-1α? Third, besides cell proliferation, migration, and invasion, what other malignant phenotypes are regulated by DLEU1/HIF-1α/CKAP2? Fourth, considering the regulation of DLEU1 on HIF-1α, what other hypoxia-related physiological and pathological processes are modulated by DLEU1? The questions mentioned above are still urgently to be explored.

## Conclusions

In summary, here we present seminal findings that DLEU1, by activating HIF-1α-induced transcription of CKAP2, promotes malignant growth and metastatic spread of breast cancer. This study not only corroborates the oncogenic roles of DLEU1, HIF-1α, and CKAP2, but also justifies the potential of targeting these molecules in the treatment of breast cancers, particularly those associated with higher malignancy and worse prognosis.

## Materials and methods

### Human tissue samples

Collection of human tissues was approved by the Ethics Committee of Henan Provincial People’s Hospital. Sixty patients diagnosed with breast cancer were recruited and signed informed consents to participate in this study. Paired cancer and tumor-free breast tissues were collected during the surgical resection. None of the patients received pre-operative treatment. Clinicopathological information, including TNM stages and status of lymph node metastasis, was summarized in Table 1.

**Table 1:** Association between DLEU1 and clinicopathological characteristics of breast cancer patients.

Variables	Cases (*n* = 60)	DLEU1		*P* value
		Low (*n* = 27)	High (*n* = 33)	
Age (years)				**0.657**
<50	33	14 (42.4%)	19 (57.6%)	
≥50	27	13 (48.1%)	14 (51.9%)	
Menopause				**0.802**
No	41	18 (43.9%)	23 (56.1%)	
Yes	19	9 (47.4%)	10 (52.6%)	
Tumor size				**0.002**^**a**^
≤2.0 cm	25	17 (68%)	8 (32%)	
å 2.0 cm	35	10 (28.6%)	25 (71.4%)	
Lymph node metastasis				**0.006**^**a**^
No	26	17 (65.4%)	9 (34.6%)	
Yes	34	10 (29.4%)	24 (70.6%)	
TNM stage				**0.013**^**a**^
I–II	23	15 (65.2%)	8 (34.8%)	
III–IV	37	12 (32.4%)	25 (67.6%)	

^a^*p* < 0.05, statistically significant.

### Bioinformatic analysis

Expressional and correlational analyses of DLEU1, HIF-1α, and CKAP2 were performed using breast cancer datasets from the Cancer Genome Atlas (TCGA; https://portal.gdc.cancer.gov/). The interaction between DLEU1 and HIF-1α was profiled using catRAPID algorithm (http://s.tartaglialab.com/page/catrapid_group). The HRE within the promoter region of CKAP2 was predicted using JASPAR platform (http://jaspar.genereg.net/).

### Colony formation assay

Target cells (200/well) were seeded into 24-well plate and cultured in growth medium for 10 days, during which, fresh medium was replaced every 3 days. Cell colonies formed were stained with 1% crystal violet (Sigma), photographed, and quantified with NIH Image J (https://imagej.nih.gov/ij/).

### Transwell assay

Transwell inserts (Corning, Lowell, MA, USA) without (for migration) or with Matrigel coating (for invasion) were used to examine motility of breast cancer cells. Briefly, target cells in serum-free medium were added into the top well (1 × 10^5^ cells/well) and serum-containing medium to the lower chamber. After 24 h, non-invading cells were removed with cotton swabs, and migrating or invading cells were stained with 1% crystal violet, imaged, and counted.

### RNA immunoprecipitation (RIP)

RIP assay was performed following the instructions from EZ-Magna RIP RNA-Binding Protein Immunoprecipitation Kit (Sigma). After incubation of the cell lysate with the anti-HIF-1α (ab1, Abcam) or normal mouse IgG (ab188776, negative control), DLEU1 in the IP complex was detected by RT-PCR. DLEU1 in total RNA was detected as the input control. The amount of DLEU1 precipitated was presented as a % of total input.

### RNA pull-down assay

Biotin-labelled RNA transcripts corresponding sense DLEU1, antisense DLEU1, and a negative control sequence were synthesized by in vitro transcription with T7 RNA polymerase and the biotin RNA labeling kit (Abcam). Purified PCR products were then incubated with whole cell lysates prepared from MDA-MB-468 or MCF7 cells and purified using Streptavidin Sepharose High Performance resin (GE Healthcare, Pittsburgh, PA, USA). Recruited HIF-1α was examined by western blot.

### Chromatin immunoprecipitation (ChIP) assays

Crosslinking of target cells was accomplished with 1% formaldehyde before cells were sonicated to generate DNA/protein fragments of approximately 500-1000 base pairs. Digested chromatin was then incubated with anti-HIF-1α antibody (ab228649) or normal rabbit IgG (negative control; both from Abcam) at 4 °C overnight, followed by protein G magnetic beads (Thermo Fisher, Waltman, MA, USA). Precipitated DNA was purified and examined with RT-PCR.

### In vivo tumor formation and metastasis assays

Four- to six-week-old female BALB/c nude mice were ordered from SJA Laboratory Animal (Hunan, China) and house in a SPF facility. To generate xenografts, shNC or shDLEU1#2 MDA-MB-468 cells were injected subcutaneously into mouse back (1 × 10^6^ cells/mouse, *n* = 6/group), within the principle of random allocation. Every 3 days thereafter, tumors were measured using a caliper. All mice were sacrificed after 18 days.

For metastasis, shNC or shDLEU1#2 MDA-MB-468 cells were injected into the tail vein (1 × 10^6^ cells/mouse, *n* = 6/group), within the principle of random allocation. After 28 days, mice were sacrificed and lungs were isolated, counted for metastatic nodules, and processed for histological analysis. Animal protocols were designed following the guidelines and received approval from the Institutional Animal Care and Use Committee of Henan Provincial People’s Hospital.

### Statistical analysis

Data analysis was accomplished with SPSS 22.0 (IBM, Chicago, IL, USA) and expressed as mean ± standard deviation (SD) from three independent in vitro experiments or from all mice in a group. All data were in a normal distribution, and variance was similar between the groups that are being statistically compared. Student’s *t* test or one-way ANOVA was used to assess differences between groups. Statistical significance was defined as *P* < 0.05.

Other methods used in this study and original western blots were shown in Supplemental materials.

## Supplementary information

fig.S1

fig.S2

Supplemental materials

## Data Availability

All data generated or analyzed during this study are included in this published paper.
