# Supplementary figures and images for: Long non-coding RNA DLEU1 promotes malignancy of breast cancer by acting as an indispensable coactivator for HIF-1α-induced transcription of CKAP2
Source: Cell Death Dis. 2022 Jul 19;13(7):625. doi: 10.1038/s41419-022-04880-z (PMC9296616; doi:10.1038/s41419-022-04880-z)

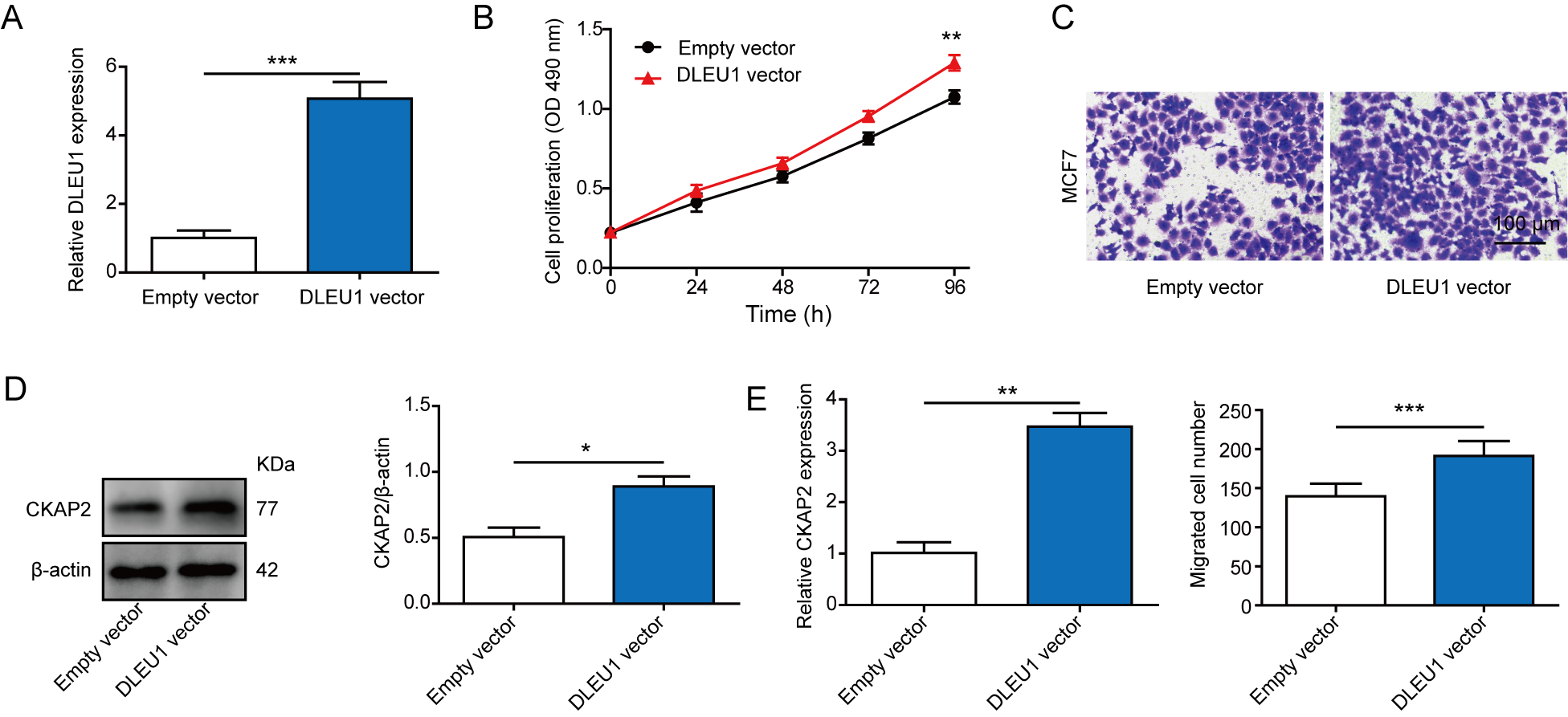

Supplement: Supplementary file 1 — fig.S1 [file 41419_2022_4880_MOESM1_ESM.tif]

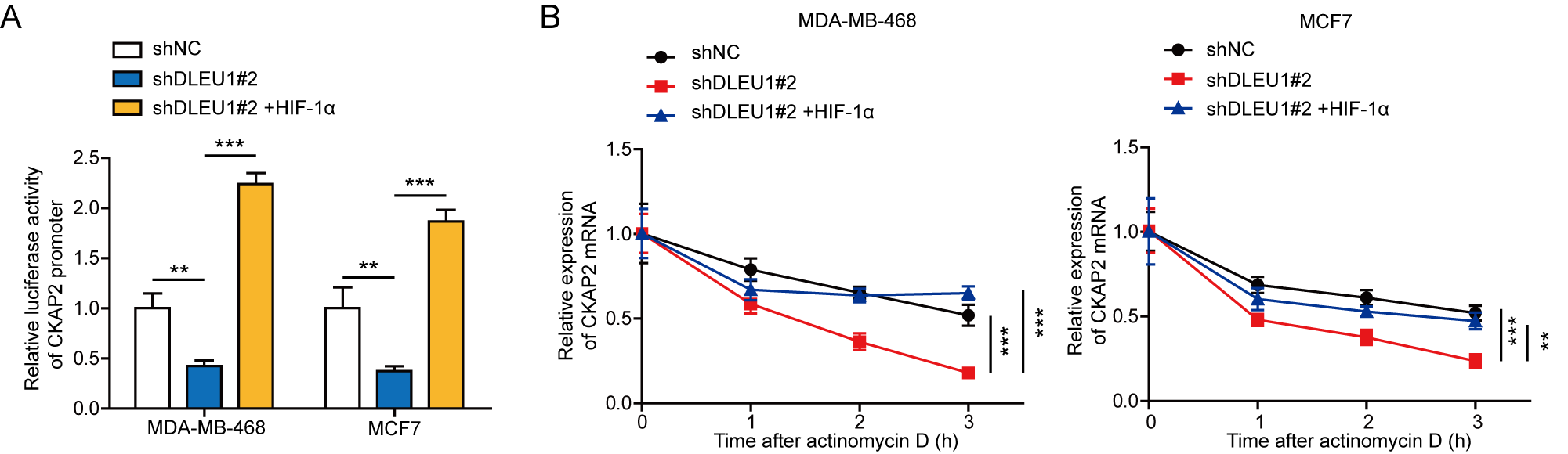

Supplement: Supplementary file 2 — fig.S2 [file 41419_2022_4880_MOESM2_ESM.tif]
