# Supplementary material for: Long non-coding RNA DLEU1 promotes malignancy of breast cancer by acting as an indispensable coactivator for HIF-1α-induced transcription of CKAP2
Source: Cell Death Dis. 2022 Jul 19;13(7):625. doi: 10.1038/s41419-022-04880-z (PMC9296616; doi:10.1038/s41419-022-04880-z)
Supplement: Supplementary file 3 — Supplemental materials [file 41419_2022_4880_MOESM3_ESM.docx]

**Supplemental Materials and Methods**

### Cell culture

Non-cancerous breast epithelial cell line MCF10A and cancerous cell lines MCF7, T47D, SK-BR-3, MDA-MB-231, MDA-MB-436, and MDA-MB-468 (American Type Culture Collection; Manassas, VA, USA) in this study had been authenticated before use. Cells were tested without contamination with mycoplasma. MCF10A cells were cultured MEBM medium supplemented with MEGM kit (Lonza, Basel, Switzerland) and 100 ng/mL cholera toxin (Sigma, St. Louis, MO, USA). Breast cancer cells were all cultured in DMEM supplemented with 10% fetal bovine serum and 1× penicillin/streptomycin (Invitrogen, Carlsbad, CA, USA).

***Cell transfection***

The pcDNA3.1 plasmid (vector), pcDNA3.1-DLEU1, pcDNA3.1-HIF-1α (HIF-1α), and pcDNA3.1-CKAP2 (CKAP2), and plasmids expressing non-targeting control shRNA (shNC), four distinct shRNAs targeting DLEU1 (shDLEU1#1, #2, #3, and #4), and two distinct shRNAs targeting HIF-1α (shHIF-1α#1 and #2) were all purchased from GenePharma (Shanghai, China). Cell transfection was performed with Lipofectamine 3000 (Invitrogen) and stable cells were established by selecting transfected cells in growth medium containing 2 µg/mL puromycin (for shRNA-expressing plasmids) and/or 600 µg/mL G418 (for pcDNA3.1 plasmids) (both from Invitrogen).

### Assay for cell proliferation

Cell proliferation was examined according to the protocol from MTT assay kit (Abcam, Cambridge, MA, USA). Briefly, indicated cells were seeded in 96-well plates (2×10^4^ cells/mL, in triplicate for each experimental condition) and cultured for 0, 24, 48, 72, or 96 h, respectively. MTT reagent was then added to and incubated with the culture medium for 3 h at 37ºC, before the plate was measured for absorbance at 490 nm.

### Luciferase reporter assay

Luciferase reporter (pRL-CMV vector) driven by either wildtype (WT) or mutant (MUT) HRE in CKAP2 gene was co-transfected into target cells with pcDNA3.1-HIF-1α vs. empty pcDNA3.1 vector. Reporter driven by CKAP2 promoter sequence was co-transfected into target cells with shNC, shDLEU1#2, or shDLEU1#2+HIF-1α. 48 h later, luciferase assay was measured following the instructions of Luciferase Reporter Assay System (Promega, Madison, WI, USA).

### mRNA stability assay and quantitative real-time PCR (RT-PCR)

To determine the stability of CKAP2, target cells were transfected with shNC, shDLEU1#2, or shDLEU1#2+HIF-1α for 48 h and then treated with actinomycin D (0.5 g/mL) for 0, 1, 2, and 3 h respectively. RNA extraction was performed using Trizol (Invitrogen) and cDNA synthesis using SuperScript III Reverse Transcriptase (Invitrogen). PCR was performed with SYBR Green Master Mix (Bio-Rad, Hercules, CA, USA). The relative expression of a target gene was normalized to that of 18s rRNA using the 2^−ΔΔCt^ method.

### Western blot

Cells were lysed in RIPA buffer (Thermo Fisher Scientific). Upon running through 10% SDS-PAGE gel, all proteins were transferred to a polyvinylidene difluoride membrane. After being incubated with primary antibodies (1:1000 dilution; all from Abcam) at 4°C overnight: anti-HIF-1α (ab1), anti-CKAP2 (ab227214), anti-p-ERK1/2 (ab223500), anti-ERK1/2 (ab184699), anti-p-STAT3 (ab76315), anti-STAT3 (ab68153), and anti-β-actin (ab6276; internal control), followed by HRP-conjugated secondary antibodies (1:2000 dilution, Abcam), target signals were developed with ECL substrate (Thermo Fisher Scientific) and normalized to that of β-actin (internal control).

***Histological analysis***

Mouse tissues were fixed with formalin and embedded into paraffin. Staining with hematoxylin and eosin (HE) was performed following the protocol from HE staining kit (Abcam). For detecting Ki-67 or CKAP2, tissue sections were boiled in citrate buffer (pH 6.0) for antigen retrieval. After blocking with 0.3% H_2_O_2_ and then with 5% normal goat serum, tissue sections were incubated with anti-Ki-67 (1:300, ab15580) or anti-CKAP2 (1:500, ab198188; both from Abcam) at 4ºC overnight and then with biotin-conjugated secondary antibody (Vector Labs). After signal amplification using Vectastain ABC-HRP (Vector Labs, Burlingame, CA, USA) solution, the color was developed with diaminobenzidine substrate (Abcam).

**Supplementary original western blots**

**
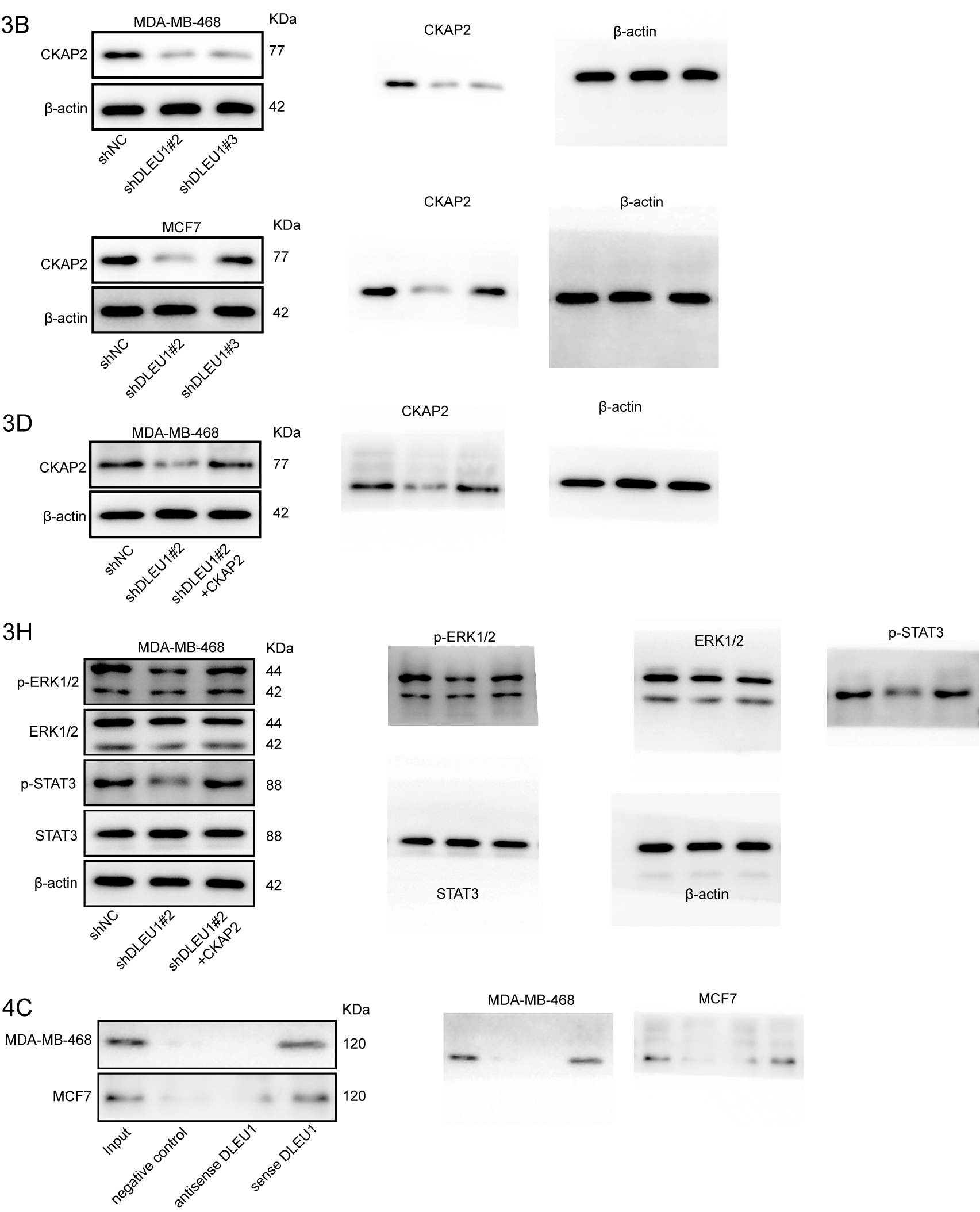
**

**
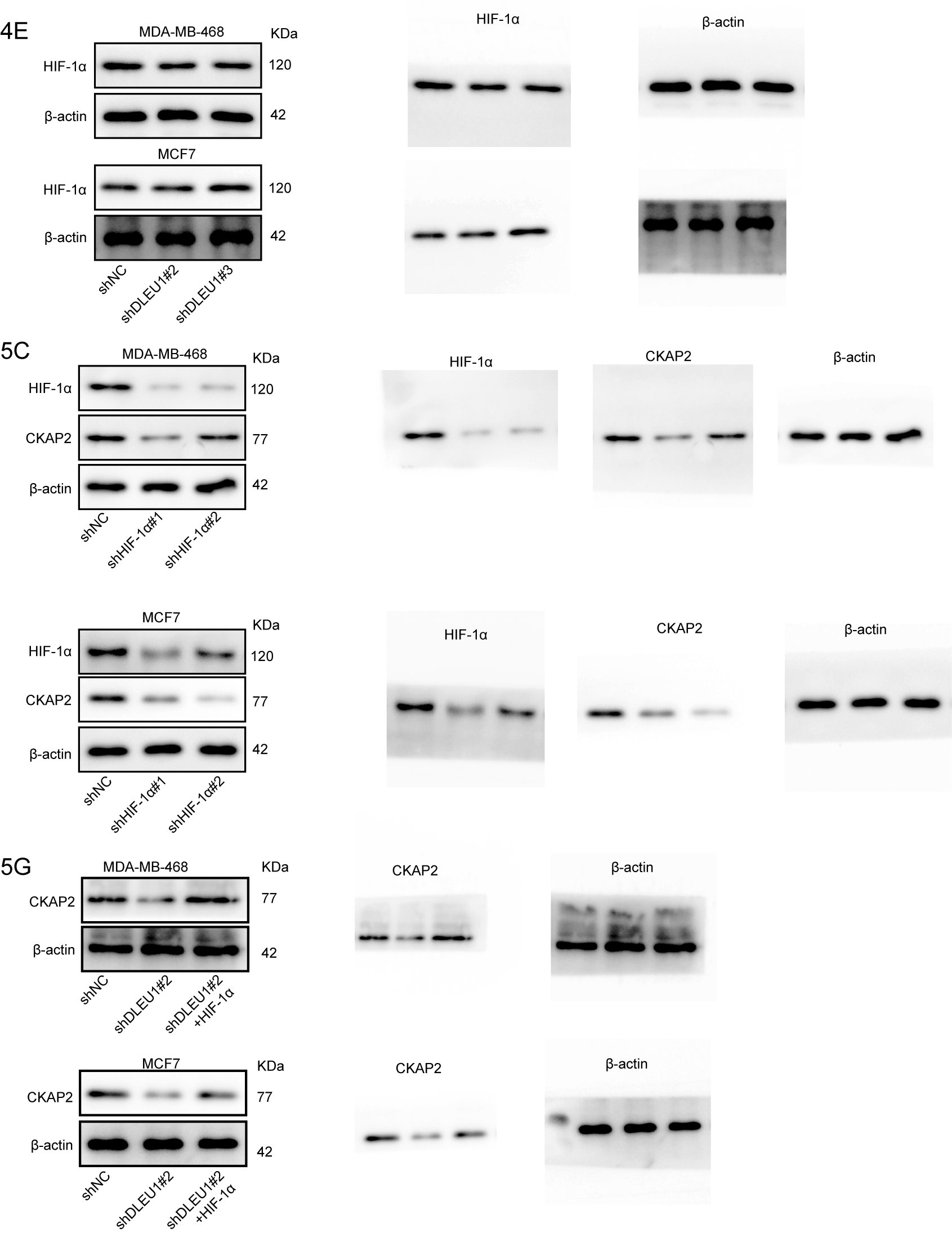
**

**
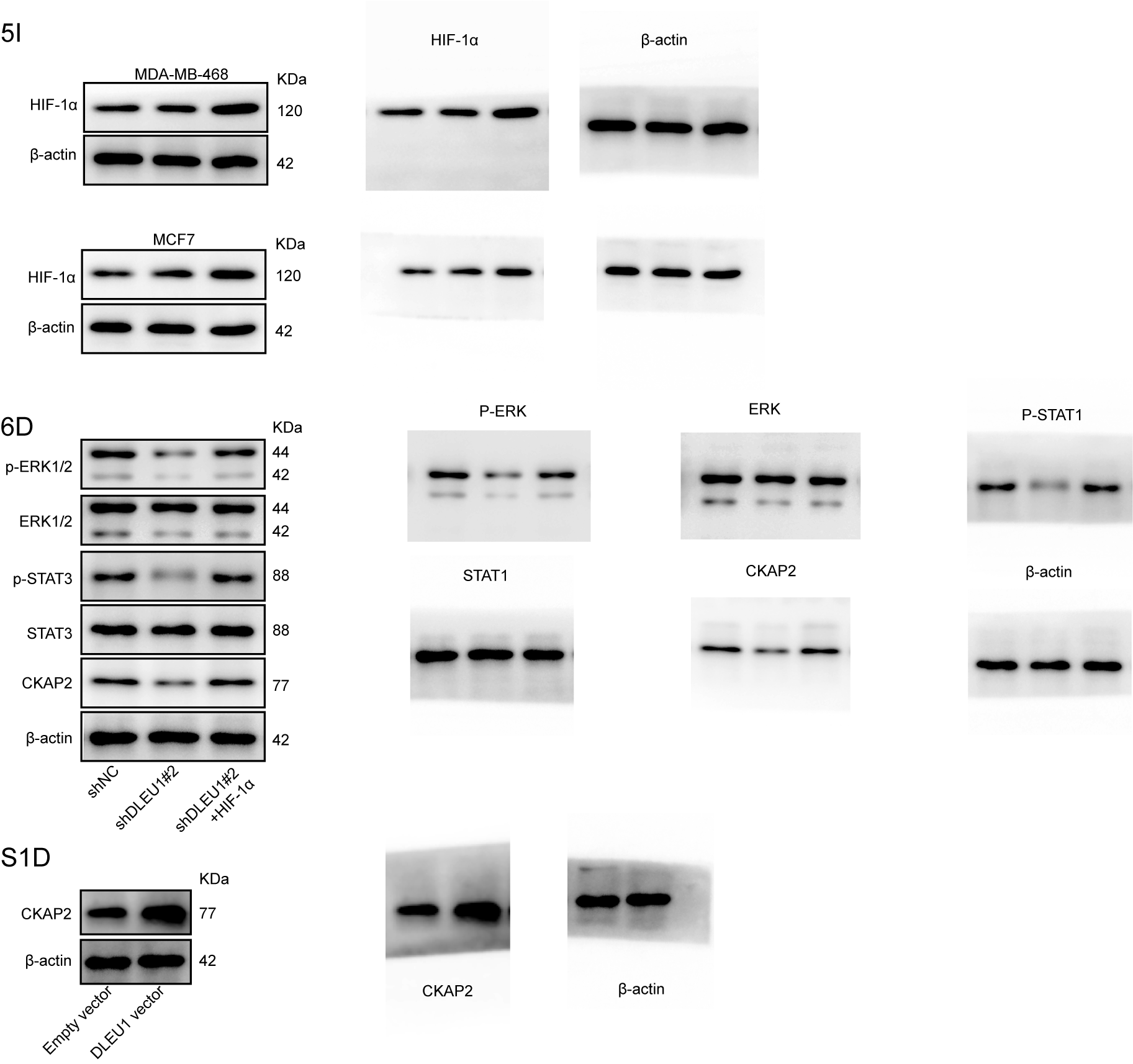
**

**Supplemental figure legends**

**Supplemental Figure S1. Overexpressing DLEU1 was sufficient to drive the malignancy and elevate CKAP2 expression of MCF7 cells.** MCF7 cells were transiently transfected with empty vector or DLEU1-expressing vector. A. DLEU1 level was examined by RT-PCR. B. The proliferation of indicated cells was examined by MTT assay. C. Cell migration was examined by Transwell assays. CKAP2 expression was examined by Western blot (D) and RT-PCR (E), respectively. *P < 0.05, **P < 0.01, ***P<0.001.

### **Supplemental Figure S2.** DLEU1 is a co-factor required for HIF-1α-activated transcription of CKAP2. A. Reporter activity driven by CKAP2 promoter was examined in indicated MDA-MB-468 and MCF7 cells. B. Indicated cells were treated with actinomycin D for indicated time periods and CKAP2 mRNA level was examined by RT-PCR. **P < 0.01, and ****P* <0.001.
